# FGF-23 and PTH levels in patients with acute kidney injury: A cross-sectional case series study

**DOI:** 10.1186/2110-5820-1-21

**Published:** 2011-06-14

**Authors:** MaryAnn Zhang, Raymond Hsu, Chi-yuan Hsu, Kristina Kordesch, Erica Nicasio, Alfredo Cortez, Ian McAlpine, Sandra Brady, Hanjing Zhuo, Kirsten N Kangelaris, John Stein, Carolyn S Calfee, Kathleen D Liu

**Affiliations:** 1 Albert Einstein College of Medicine, Yeshiva University, New York, NY, USA; 2 Division of Nephrology, Department of Medicine, University of California, San Francisco, CA, USA; 3 Cardiovascular Research Institute, University of California, San Francisco, USA; 4 Department of Emergency Medicine, University of California, San Francisco, CA, USA; 5 Division of Hospital Medicine, Department of Medicine, University of California, San Francisco, CA, USA; 6 Division of Pulmonary and Critical Care Medicine, Department of Medicine, University of California, San Francisco, CA, USA

## Abstract

**Background:**

Fibroblast growth factor-23 (FGF-23), a novel regulator of mineral metabolism, is markedly elevated in chronic kidney disease and has been associated with poor long-term outcomes. However, whether FGF-23 has an analogous role in acute kidney injury is unknown. The goal of this study was to measure FGF-23 levels in critically ill patients with acute kidney injury to determine whether FGF-23 levels were elevated, as in chronic kidney disease.

**Methods:**

Plasma FGF-23 and intact parathyroid hormone (PTH) levels were measured in 12 patients with acute kidney injury and 8 control subjects.

**Results:**

FGF-23 levels were significantly higher in acute kidney injury cases than in critically ill subjects without acute kidney injury, with a median FGF-23 level of 1948 RU/mL (interquartile range (IQR), 437-4369) in cases compared with 252 RU/mL (IQR, 65-533) in controls (*p *= 0.01). No correlations were observed between FGF-23 and severity of acute kidney injury (defined by the Acute Kidney Injury Network criteria); among patients with acute kidney injury, FGF-23 levels were higher in nonsurvivors than survivors (median levels of 4446 RU/mL (IQR, 3455-5443) versus 544 RU/mL (IQR, 390-1948; *p *= 0.02). Severe hyperparathyroidism (defined as intact PTH >250 mg/dL) was present in 3 of 12 (25%) of the acute kidney injury subjects versus none of the subjects without acute kidney injury, although this result did not meet statistical significance.

**Conclusions:**

We provide novel data that demonstrate that FGF-23 levels are elevated in acute kidney injury, suggesting that FGF-23 dysregulation occurs in acute kidney injury as well as chronic kidney disease. Further studies are needed to define the short- and long-term clinical effects of dysregulated mineral metabolism in acute kidney injury patients.

## Introduction

Acute kidney injury (AKI) is the most common reason for inpatient nephrology consultation and is associated with in-hospital mortality rates of 45-70% [1,2]. Until recently, studies of AKI have focused on the epidemiology and management of AKI during the index hospitalization. However, AKI is now recognized as a disease with long-term sequelae, including increased risk of death and chronic kidney disease (CKD) progression [3-10]. The mechanisms by which AKI is linked to adverse long-term outcomes are poorly understood. Changes commonly found in CKD patients--anemia, acid/base dysregulation, altered mineral metabolism--likely occur in AKI patients, and as in CKD patients, may be responsible for some of these adverse long-term sequelae.

Dysregulated mineral metabolism, including derangements in calcium and phosphate levels, is relatively well characterized in CKD, and correction of hypocalcemia, vitamin D deficiency, and hyperphosphatemia in CKD patients is standard-of-care [11-13]. These derangements are all associated with an increased risk of death and cardiovascular outcomes in patients with CKD and end-stage renal disease [14-22]. Interestingly, although hypocalcemia and hyperphosphatemia are commonly observed in patients with AKI, the literature on dysregulated mineral metabolism in this patient population is relatively limited. Some papers have concentrated on rhabdomyolysis-induced AKI, where hyperphosphatemia is extreme due to tissue breakdown [23-30]. Several studies included patients with AKI due to causes other than rhabdomyolysis [23,30-35], but these were published 30 or more years ago and did not measure more novel regulators of mineral metabolism, such as fibroblast growth factor-23 (FGF-23).

FGF-23 is a 26-kD protein that is a novel, key regulator of phosphorus excretion and contributes to abnormal bone metabolism in CKD [36]. FGF-23 has been shown to be a strong, independent predictor of death in ESRD and CKD [37-39]. To date, only one case report has explored the impact of AKI on levels of FGF-23 and that was in the setting of rhabdomyolysis [40]. We sought to determine the impact of AKI on FGF-23 and parathyroid hormone (PTH) levels in patients with AKI due to causes other than rhabdomyolysis. If FGF-23 is elevated in this context, we hypothesized that FGF-23 might represent a novel treatment target or a novel predictor for poor outcomes in patients with AKI.

## Methods

### Study design, patient selection, and clinical data collection

AKI cases and non-AKI control subjects (controls) were selected from two prospective observational cohort studies conducted at a tertiary care university hospital. Cases were identified from a prospective study of all patients with AKIN Stage I AKI [41] admitted to the adult intensive care unit of University of California San Francisco Medical Center between June 2006 and March 2009. Control subjects without AKI were identified from a prospective study of all critically ill Emergency Department patients eligible for admission to the adult intensive care unit of University of California San Francisco Medical Center from October 2008 to the present. The protocols were approved by the Institutional Committee on Human Research.

Baseline creatinine was defined as the lowest creatinine from the 365 days before admission until the episode of AKI for AKI subjects. For control subjects, baseline creatinine were the lowest creatinine from the 365 days before admission until hospital admission. Potential subjects with a baseline creatinine of greater than 1.1 mg/dL were excluded to eliminate subjects with underlying CKD, which would impact FGF-23 and PTH levels. Cause of AKI was determined by two nephrologists (KDL, RH) based on chart review. Each nephrologist independently reviewed the medical record to determine the cause of AKI; there was 100% agreement between the two reviewers.

### Biomarker measurements

Plasma samples obtained from cases and controls were immediately spun at 3000 rpm for 10 minutes and were aliquoted and stored at -80°C until biomarker measurements were made. For AKI cases, samples were obtained at regular intervals during the first week that the patient met criteria for AKI; measurements were made on the sample from the time point closest to the peak serum creatinine. For controls, measurements were made on samples obtained immediately after admission. Intact PTH measurements were made using Immulite 2000 Intact PTH assay (Siemens, Deerfield IL). FGF-23 measurements were made using a C-terminal FGF-23 ELISA (Immutopics, San Clemente, CA) according to the manufacturer's instructions. The reported calcium and phosphorus measurements were made as part of routine clinical care; the reported measurements are the closest available relative to the time of biosample collection.

### Statistical analyses

Baseline characteristics of cases and controls were first compared. Categorical variables were expressed as proportions, and compared using the χ^2 ^test. Continuous variables were expressed as mean ± standard deviation or median with interquartile range and were compared using the *t *test or the Mann-Whitney rank-sum test, where appropriate. Spearman rank correlation coefficients were used to correlate FGF-23 levels with serum phosphorus, calcium, and PTH levels. Linear regression analysis was used to examine the relationship between FGF-23 levels and AKI status, after controlling for age and severity of illness, as measured by Acute Physiology And Chronic Health Evaluation (APACHE) II score [42]; because FGF-23 levels were not normally distributed, levels were natural log transformed for this analysis. Data analysis was conducted by using Stata 10.1 (StataCorp, College Station, TX). Two-tailed *p *values < 0.05 were considered significant.

## Results

The baseline demographics and clinical characteristics of the 20 subjects in this study are summarized in Table 1. We studied 12 cases who developed at least Stage I AKI and 8 control subjects who did not. Cases and controls were similar with regard to sex and race. On average, cases were younger than controls (57 ± 12 years versus 70 ± 17 years, *p *= 0.05) and had lower APACHE II scores (27 ± 11 versus 17 ± 8, *p *= 0.04). There was no statistically significant difference for in-hospital mortality rates between the two groups.

**Table 1:** Baseline characteristics of patients with and without acute kidney injury

	No acute kidney injury	Acute kidney injury	*p *value
Number	8	12	
Age (yr)*	70 ± 17	57 ± 12	0.05
Male n (%)	2(25%)	6(50%)	0.37
Caucasian n (%)	4(50%)	10(83%)	0.16
APACHE II*	17 ± 8	27 ± 11	0.04
Death n (%)	3(38%)	4(33%)	1.00
Baseline Cr (μmol/L)*	69 ± 20	67 ± 15	0.83
Peak Cr (mg/dL)*	81 ± 17	217 ± 86	<0.001
Dialysis n (%)	0(0%)	4(33%)	0.11
Ionized calcium (mmol/L)*	1.15 ± 0.08	1.19 ± 0.1	0.41
Phosphorous (mmol/L)*	3.3 ± 1.1	4.5 ± 1	0.02

*Mean ± SD

To convert Cr in μmol/L to mg/dL, divide by 88.4

Subjects with AKI had a baseline serum creatinine of 67 ± 15 μmol/L with a peak inpatient serum creatinine of 217 ± 86 μmol/L compared with a baseline serum creatinine of 69 ± 20 μmol/L and a peak serum creatinine of 81 ± 17 in non-AKI subjects (p < 0.001 for peak levels). As noted earlier, we excluded potential study subjects with a baseline creatinine greater than 97 μmol/L to avoid patients with underlying CKD. No patient had AKI attributable to rhabdomyolysis. Eight patients had acute tubular necrosis, two patients had AKI after orthotopic liver transplantation, one had cardiorenal syndrome, and one had multifactorial AKI. Two patients had Stage I AKI by the AKIN criteria [41], five patients had Stage II AKI, and five patients had Stage III AKI. Four of 12 (33%) of the AKI subjects were treated with dialysis.

**Figure 1:**
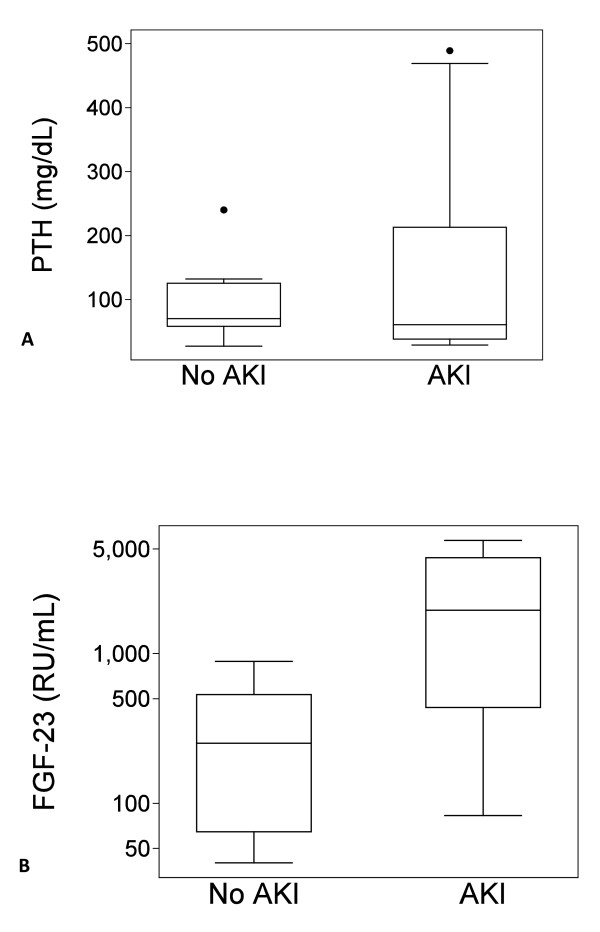
**PTH and FGF-23 levels in non-AKI and AKI subjects**. **A **There was no overall difference in PTH levels between AKI and non-AKI subjects (*p *= 0.73). **B **FGF-23 levels were significant higher in patients with AKI compared with non-AKI subjects (*p *= 0.01).

Subjects with and without AKI had mean ionized calcium levels of 1.19 ± 0.1 mmol/L and 1.15 ± 0.08, respectively (*p *= 0.41). Serum phosphorus levels were significantly higher in AKI subjects compared with controls (4.5 ± 1 mmol/L versus 3.3 ± 1.1 mmol/L, *p *= 0.02). The median intact PTH level was 63 mg/dL (25-75% interquartile range (IQR), 38-213) in AKI subjects and 70 mg/dL (25-75% IQR, 58-126) in controls (Figure 1A, *p *= 0.73). When severe hyperparathyroidism was defined as an intact PTH >250 mg/dL, a level that has been associated with increased cardiovascular disease risk in prior studies [43], none of the control subjects had a severe hyperparathyroidism but 3 of 12 (25%) of the AKI subjects did (although this result did not meet conventional levels of statistical significance, *p *= 0.24).

**Figure 2:**
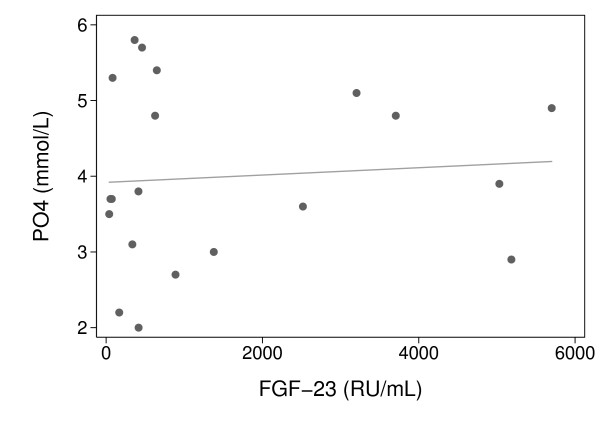
**Correlation of serum phosphorus (PO4) and FGF-23 levels in patients with and without acute kidney injury**. No correlation was observed between PO4 and FGF-23 levels (r = 0.08, *p *= 0.74).

FGF-23 levels were significantly higher in critically ill AKI cases compared with critically ill non-AKI subjects, with a median FGF-23 level of 1948 RU/mL (IQR, 437-4369) in AKI cases compared with 252 RU/mL (IQR, 65-533) in critically ill controls (*p *= 0.01; Figure 1B). After adjusting for age and APACHE II as potential confounders, AKI remained a significant predictor of log-transformed FGF-23 levels (Table 2). Among patients with AKI, FGF-23 levels were higher in nonsurvivors (n = 4) compared with survivors (n = 8), with respective median levels of 4446 RU/mL (IQR, 3455-5443) versus 544 RU/mL (IQR, 390-1948; *p *= 0.02). Although serum phosphorus and FGF-23 levels were both elevated in AKI subjects, no correlation was observed between the two variables, as shown in Figure 2 (r = 0.08, *p *= 0.74). There was a correlation between PTH and FGF-23 levels (r = 0.55, *p *= 0.02); when this analysis was restricted to patients with AKI, this correlation only had borderline statistical significance (r = 0.58, *p *= 0.05), likely due to the small size of the cohort.

**Table 2:** Association of log-transformed FGF-23 levels with AKI (multivariable linear regression)

Predictor	Coefficient	95% CI	*p *value
AKI	1.81	0.37-3.25	0.02
Age*	0.29	-0.15-0.73	0.18
APACHE II score	0.05	-0.02-0.11	0.14

*Per 10-year increase

## Discussion

In this cross-sectional case series, we report for the first time that critically ill patients with AKI due to causes other than rhabdomyolysis have elevated FGF-23 levels compared with critically ill controls. Among patients with AKI, elevated FGF-23 levels were associated with an increased risk of death. As expected, AKI patients had, on average, higher concentrations of serum phosphorous compared with patients without AKI. In addition, a larger proportion of AKI patients had significant hyperparathyroidism compared with controls, although this result did not meet statistical significance. These results suggest that dysregulated mineral metabolism is common in AKI, analogous to CKD.

Interestingly, no correlation was observed between phosphorous and FGF-23 levels in this study. Few reports have analyzed this relationship in AKI patients, although in ESRD a patient's degree of elevation in FGF-23 is often correlated with severity of hyperphosphatemia [38,44,45]. In CKD, elevated FGF-23 levels are thought to be due to increased secretion by bone cells, rather than due to decreased renal clearance [46,47]. Comparison of levels of intact versus degraded FGF-23 in patients on maintenance hemodialysis suggest that there is no increase in FGF-23 degradation products in these subjects and that decreased clearance of FGF-23 is therefore not the mechanism for increased FGF-23 levels [48]. Therefore, as in CKD, elevated FGF-23 levels in AKI are likely not due to decreased clearance of FGF-23 and highlight the important paracrine role of the kidney, even in an acute illness (e.g., AKI).

Elevation of FGF-23 during AKI may have several implications. In ESRD patients undergoing hemodialysis, high FGF-23 concentrations are associated with early mortality, with an increased risk as high as 600% [37,38]. Our study demonstrated an association between FGF-23 levels and death in subjects with AKI, although relatively small. If the association between elevated FGF-23 levels and death is confirmed in a larger study of patients with AKI, prevention or treatment of such processes could become a priority in AKI management. Treatment with phosphate binders and calcimimetics (Cinacalcet) has been shown to lower FGF-23 levels [49-51]. Treatments that are tailored more toward AKI-induced mineral dysregulation could be developed as further information is gathered about the exact role of FGF-23 in AKI. At present, there are no evidence-based guidelines about target goals for maintaining serum phosphorus levels. Treatments that improve the long-term outcomes of patients with AKI are needed, and dysregulated mineral metabolism, including FGF-23 levels, may represent a therapeutic target in AKI that is highly amenable to intervention.

There are several limitations in this study, including small sample size and relatively short follow-up time. Because FGF-23 levels were not measured repeatedly, duration of FGF-23 elevation also was unclear. Nevertheless, this is the first study to report an association between FGF-23 and non-rhabdomyolysis-related AKI, and that among patients with AKI, higher FGF-23 levels are associated with an increased risk of death. Larger and long-term studies should be conducted to clarify the impact of FGF-23 elevation among AKI patients.

## Conclusions

Dysregulated mineral metabolism is a poorly understood aspect of acute kidney injury. We demonstrated for the first time that FGF-23, a critical regulator of mineral metabolism in chronic kidney disease, is upregulated during acute kidney injury from causes other than rhabdomyolysis. Furthermore, high FGF-23 levels are associated with mortality in patients with AKI.

## Competing interests

The authors declare that they have no competing interests.

## Authors' contributions

MZ and RH were responsible for data analysis and manuscript preparation. CYH was responsible for study design, data analysis, and manuscript preparation. KK, EN, AC, and IA were responsible for the execution of the study, including screening and consenting eligible study subjects, data collection, data analysis, and manuscript preparation. HZ was responsible for database management and data analysis. SB was responsible for FGF-23 measurements. KNK, JS, and CSC were responsible for design of the study and manuscript preparation. KDL was responsible for study design, biomarker measurements, data analysis, and manuscript preparation.
